# A functional crosstalk between circulating follicular helper 2 T cells and memory B cells drives anti-*Plasmodium vivax* antibodies

**DOI:** 10.1371/journal.pntd.0014232

**Published:** 2026-04-24

**Authors:** Zulfa Zahra Salsabila, Piyawan Kochayoo, Tipanan Khunsri, Pachara Tianpothong, Chaniya Leepiyasakulchai, Pongsakorn Thawornpan, Julius Clemence R. Hafalla, John H. Adams, Patchanee Chootong

**Affiliations:** 1 Department of Clinical Microbiology and Applied Technology, Faculty of Medical Technology, Mahidol University, Bangkok, Thailand; 2 Department of Community Medical Technology, Faculty of Medical Technology, Mahidol University, Bangkok, Thailand; 3 Department of Infection Biology, London School of Hygiene and Tropical Medicine, London, United Kingdom; 4 Center for Global Health and Inter-Disciplinary Research, College of Public Health, University of South Florida, Tampa, Florida, United States of America; University of Antwerp Drie Eiken Campus: Universiteit Antwerpen Campus Drie Eiken, BELGIUM

## Abstract

**Background:**

The induction of long-term humoral immune responses depends upon the interaction between T follicular helper (Tfh) and B cells in germinal centers, leading to development of memory B cells (MBCs) and class-switched antibodies. Expansion and activation of circulating Tfh2 (cTfh2) cells were detected in both vivax and falciparum malaria subjects. However, how these cells help B cells generate anti-malarial immunity is still unclear. Here, we assessed the breadth and competency of antibody responses from *P. vivax* subjects and related them to the frequency and activation status of cTfh2 subset. We also demonstrated the ability of *P. vivax* antigen to trigger cTfh2 cell activation and the function of cTfh2 cells to help MBCs secrete antibodies.

**Methodology/Principal findings:**

Of 40 subjects with acute *P. vivax* malaria, 23 were seropositive for anti-PvDBPII antibodies (High and Low responders). Three High Responders (HRs) produced inhibitory antibodies against PvDBPII-human erythrocyte binding. An expansion of cTfh2 cells was detected in seropositive subjects. While their frequency did not differ significantly between High and Low Responders (LRs), the expression of co-stimulatory molecule ICOS in cTfh2 cells was higher in HRs. Activation of cTfh2 cells was specifically stimulated by PvDBPII antigen. In cTfh2-MBC co-cultures, proliferation and activation of cTfh2 cells were detected after receiving signal from MBCs. These activated cTfh2 cells then promoted MBC differentiation into antibody secreting cells (ASCs) which secreted anti-PvDBPII IgG. A decrease in cTfh2 cell activation was observed upon the addition of IFN-γ to the co-cultures. Importantly, cTfh2 cells played a role in producing anti-malarial specific antibodies.

**Conclusions/Significance:**

This study demonstrated that activation of cTfh2 cells, marked by the upregulation of ICOS molecules, was notably observed in subjects who produced high titers of anti-PvDBPII antibodies in response to *P. vivax* infection. This was also seen in a few subjects who produced high levels of antibody with inhibitory function. The PvDBPII antigen specifically stimulated cTfh2 cell proliferation and activation. Additionally, interactions between cTfh2 cells and MBCs promoted both cTfh2 activation and anti-PvDBPII antibody secretion.

## Author summary

Naturally acquired immunity to malaria relies on humoral immunity which plays a crucial role in inhibiting parasite invasion. Interactions between Tfh and B cells in germinal centers contribute to MBC development and antibody production. Although an expansion of cTfh2 cells has been reported during acute malaria, the mechanism by which Tfh2 cells support the development of MBCs and antibodies is poorly understood. Here, the activation of cTfh2 cells was detected in *P. vivax* subjects with high levels of anti-PvDBPII IgG antibodies and also a few who produced inhibitory antibodies. Proliferation and activation of cTfh2 cells were specifically stimulated by *P. vivax* antigen. Upon cTfh2-MBC co-culture, cTfh2 cells proliferated and activated, while MBCs differentiated into plasma cells and secreted antibodies. Our study provides data on the role of Tfh2 cells in the development of antibodies specific to *P. vivax* antigen.

## Introduction

*Plasmodium vivax* is the most geographically widespread species and the second largest contributor to clinical malaria worldwide [1,2]. It presents unique challenges for malaria control due to the parasites’ having the ability to form dormant liver stages (hypnozoites) which can cause relapses long after the initial infection [3,4]. The high genetic polymorphism of *P. vivax* parasite enhances antigenic variation, contributes to re-infection and limits the effectiveness of naturally acquired immunity which leads to short-lived immune responses [4,5]. To control or eradicate malaria, a highly efficacious vaccine is needed to protect from or reduce the severity of clinical malaria and disrupt its transmission. To date, protective effects of malaria (RTS,S/AS01) vaccines have been shown to be short-lived, depending on the intensity of transmission of specific endemic areas [6–9]. Thus, it is essential to better understand the mechanisms underlying the generation and maintenance of memory B cells (MBCs) and long-lived plasma cells (LLPCs) in malaria.

The induction of long-term humoral immune responses depends upon formation of a productive germinal center (GC) where B cells differentiate into MBCs and produce high-affinity antibodies [10]. In GC responses, follicular helper T (Tfh) cells provide help to B cells via multiple pathways, including secretion of cytokines (IL-21 and IL-4) and expression of co-stimulatory molecules (ICOS, CD40L and PD-1), resulting in development of MBCs and switched class antibodies [11,12]. Thus, induction of Tfh cell function is a key player in generating a durable humoral immune response. To study circulating Tfh (cTfh) cells, CD4^+^CD45RA^-^T cells expressing CXCR5 are categorized into distinct cytokine-producing subsets based on CXCR3 and CCR6 expression: CXCR3^+^CCR6^-^ (cTfh1), CXCR3^-^CCR6^-^ (cTfh2), CXCR3^-^CCR6^+^ (cTfh17), and CXCR3^+^CCR6^+^ (cTfh1/17) [13–15]. These subsets are transcriptionally different and produce distinct cytokines to regulate the humoral responses that are crucial to further characterizing the role of each cTfh subset within the context of their interactions with B cells to promote antibody production.

To date, knowledge of the mechanisms of cTfh cells in cooperative interaction with B cells for producing anti-malaria humoral immunity is limited. Among four subsets of cTfh cells (cTfh1, cTfh2, cTfh17, and cTfh1/17), an elevation of cTfh2 cells during *P. falciparum* infection was shown in the Controlled Human Malaria Infection (CHMI) study [16]. Similarly, in *P. vivax* studies, an expansion of cTfh2 cells was detected in acutely-infected subjects, and its frequency was positively association with anti-malarial antibodies [17–19]. The polarization of cTfh2 cells was positively related to the frequency of both atypical MBCs (CD21^-^CD27^-^) and plasmablasts [18]. These findings demonstrate that natural malaria infection triggers activation of cTfh2 cells that is related to humoral immune responses. However, the precise mechanisms underlying the signaling pathway of cTfh2 cell activation, and their role in providing help to B cells in the generation of antibodies against malaria is still lacking. A deep investigation of how cTfh2 cells contribute to B cell activation, and the generation and maintenance of MBCs should be helpful in the development of effective malaria vaccines.

Here, we aimed to investigate the function of cTfh2 cells in helping MBC responses for generation of antibodies following *P. vivax* infection. To do so, we assessed the breadth and function of antibodies in *P. vivax*-infected subjects and studied the correlations among antibody titers, their inhibitory function, and the frequency of cTfh2 cells in individuals. *In vitro* cultures were used for analysis of cTfh2 responses, signals that drive co-stimulatory capacities and functions of cTfh2 cells in cooperation with MBCs for promoting antibody secretion. Together, our findings provide greater understanding of cTfh2 cell function which may allow more efficacious malaria vaccine design.

## Materials and methods

### Ethics statement

Written informed consent was obtained from each study participant. This study was approved by Mahidol University Central Institutional Review Board (MU-CIRB 2021/281.2505). All experiments involving human subjects were conducted following relevant guidelines and regulations.

### Study site and population

Blood samples were collected from *P. vivax* subjects who lived in a malaria low-transmission area in the Southern part of Thailand (Chumphon Province), where both *P. falciparum* and *P. vivax* were common. The *P. vivax* subjects were recruited and enrolled at malaria clinics (Vector Borne Disease Units 11.4) from June to September 2024. The age of subjects ranged from 18 to 63 years old. For study inclusion, they had systolic blood pressures greater than 90 mmHg, body temperatures lower than 40^o^C, and hematocrits higher than 25%. The seven recovery subjects (n = 7) who were diagnosed with *P. vivax* infection at a malaria clinic and they had been recovered from malaria for 7–8 months were recruited. They were visited at home weekly by malaria clinic staff to estimate the incidence of clinical malaria infections during the study period. Forty symptomatic subjects were enrolled and donated blood samples to assess IgG, and IgG subclasses, antibodies specific to *P. vivax* Duffy Binding Protein II (PvDBPII) antigen. Seropositive subjects were classified as High Responders (HRs; n = 9) or Low Responders (LRs; n = 14), then they were also assessed for inhibitory antibodies against PvDBPII-erythrocyte binding, and characterized as to cTfh cell subsets. The ability of PvDBPII to stimulate cTfh2 cell activation was demonstrated in acute (n = 7) and recovered (n = 7) subjects. Of the nine HR subjects, six were studied for co-operative role of cTfh2 and MBCs using *in vitro* co-cultures.

Acute symptomatic *P*. *vivax* infections were each documented microscopically using both thin and thick, Giemsa-stained blood smears, and then confirmed by nested PCR [20]. Briefly, genomic DNA was amplified in a 20 µL primary PCR using primers rPLU6_F (TTA AAA ATT GTT GCA GTT AAA ACG) and rPLU5_R (CCT GTT GTT GCC TTA AAC TTC). Cycling conditions were 95°C for 4 min, followed by cycles of 95°C for 1 min, 55°C for 1 min, and 72°C for 1 min. The primary amplicon was used as a template for nested PCR with *P. vivax*–specific primers rVIV1_F (CGC TTC TAG CTT AAT CCA CAT AAC TGA TAC) and rVIV1_R (ACT TCC AAG CCG AAG CAA AGA AAG TCC TTA), using identical reaction conditions except for an annealing temperature of 60°C. PCR products were resolved on a 2% agarose gel stained with a fluorescent nucleic acid dye.

Venous blood samples were collected in heparinized vacutainer tubes (Becton Dickinson) and transported to the laboratory within 4–6 hours. Data on past malaria infections were obtained from the records kept by the local malaria clinics and Vector Borne Unit 11.4. In addition, blood samples were taken from healthy controls (HCs; n = 21) who lived in Bangkok, a non-endemic area of Thailand. The demographic information of recruited subjects is summarized in **S1 Table**.

### ELISA detection of total IgG and IgG subclasses

The plasma from *P. vivax-*acutely patients and HCs were tested for anti-PvDBPII antibody titers using an indirect ELISA, as previously reported [18]. Briefly, 96-well microtiter plates were coated with 2 µg/ml recombinant PvDBPII (rPvDBPII) protein and incubated overnight at 4°C. After blocking, diluted plasmas (1:200) were added to duplicate wells and incubated for 1 h. HRP-conjugated, goat anti-human IgG or IgG subclasses (Seracare Life Sciences, USA) was then added to the wells and incubated for 1 h, followed by addition of TMB (Merck Millipore, Germany) to detect antigen-antibody reactivity. A Multiscan SkyHigh Microplate Reader (Thermo Fisher Scientific, USA) was used to determine the absorbance at 450 nm. The levels of total IgG and IgG subclasses were standardized as a reactivity index (RI), calculated by dividing OD values of tested samples by a cut-off value (mean + 2SD) based on HC samples. An RI greater than or equal to 1.0 was considered seropositive, and an RI less than 1.0 was considered seronegative. The seropositivity of total IgG was classified into 2 groups. An RI value greater than or equal to the cut-off + 2SD was classified as a high responder (HRs), an RI greater than the cut-off but less than the cut-off + 2SD was classified as a low responder (LRs). For seronegative subjects who showed RI lower than the cut-off value was classified as a non-responder (NRs).

### COS7 inhibition erythrocyte binding assay

Expression plasmid constructs were engineered to target PvDBPII alleles on the surface of transiently transfected COS7 cells as fusion proteins to the N-terminus of enhanced green fluorescent protein (EGFP). PvDBPII-erythrocyte binding inhibition assays (EBIA) were performed as previously described [21,22]. Briefly, duplicate wells of transfected COS7 cells were pre-incubated with diluted plasma (1:200) prior to the addition of 10% Duffy-positive human erythrocytes. Rosettes were counted in at least 30 microscopic fields of view at 20x magnification. Inhibitory function was calculated as the percentage of rosettes in the presence of the tested antibody test wells relative to negative control wells. Antibody with inhibitory activity greater than or equal to 80% was considered as highly inhibitory [23–25]. 2D10 mouse monoclonal anti-PvDBPII antibody [26] and HC plasma were used as positive and negative controls of erythrocyte inhibition, respectively. The EBIA experiment was performed in duplicate and repeated twice.

### Cell staining and flow cytometry

Cryopreserved PBMCs were thawed using RPMI 1640 (Gibco, USA) containing 10% Fetal Bovine Serum (FBS) (Gibco, USA). Viable cells in each sample were counted using Trypan blue (Gibco, USA) staining, and more than >95% viability was used for flow cytometric analysis. Cells were stained with monoclonal antibodies (mAbs) according to manufacturers’ recommendations (S2 Table). The characteristics of cTfh cells were determined by surface and intracellular staining with mAbs against CD3, CD4, CXCR5, PD-1, CXCR3, CCR6, ICOS, CD45RA, CD40 ligand (CD40L), TIGIT, IL-4, and IL-10. Analyses were done with a FACSymphony A1 (BD Bioscience, USA); FACS data were examined using FlowJo software version 10.10.0 and presented as cell percentages or mean fluorescence intensity (MFI).

### PBMC culture stimulation

To assess the activation of cTfh2 cells in response to *P. vivax* antigen, PBMCs stimulation assay was performed previously described [27]. Fresh PBMCs (5 x 10^5^) from *P. vivax* subjects and HCs were cultured in 96-well flat-bottomed, tissue culture plates (Corning, USA) in RPMI 1640 medium containing 10% FBS (Gibco, USA). Cells were stimulated with 100 µl of rPvDBPII (10 µg/ml) for 6 days at 37°C with 5% CO_2_. Then, frequency of cTfh cells and their cTfh2 subset, along with an expression of ICOS, were stained and acquired on a FACSCanto II Flow Cytometer (BD Biosciences). The stimulation index (SI) was calculated as the ratio of the frequency of cells in PvDBPII-stimulated cultures to that in unstimulated cultures. The positive controls were PBMCs after 1% (v/v) phytohemagglutinin (PHA) stimulation (Thermo Fisher Scientific, USA). Unstimulated PBMCs were defined as negative controls.

### cTfh2-MBC co-cultures

To develop the cTfh2-MBC co-cultures, cTfh2-like cells were generated in *in vitro* study by modifying methods from previous studies [28,29]. Naïve CD4^+^T cells (CD3^+^CD4^+^CD45RA^+^) were sorted using FACSMelody (BD Biosciences, USA). These cells were cultured in 96-well plates in the presence of 10 ng/mL anti-CD28/49d mAbs (Biolegend, USA), 20 ng/mL IL-21 (Peprotech, USA), 20 ng/mL IL-6 (Peprotech, USA), and 40 ng/mL IL-4 (Peprotech, USA) at 37°C, 5% CO_2_ for 5 days. Cells were harvested to determine phenotype of cTfh2 cells. Then, autologous MBCs were added and co-cultured in anti-CD28/49d mAbs (10 ng/mL), IL-21 (20 ng/mL), and BAFF (20 ng/mL) (Peprotech, USA). To demonstrate signals that help cTfh2-MBC interactions, the cTfh2-like cells and MBCs were co-cultured in 4 conditions: Stimuli 1 (S1) -- rPvDBPII (40 ng/mL); S2 -- rPvDBPII (40 ng/mL) and R848 (Invivogen, USA) (1 µg/mL); S3 -- rPvDBPII (40 ng/mL) and IFN-γ (Invivogen, USA) (20 ng/mL); S4 -- negative control (without stimuli). The positive control was cells with 1% (v/v) PHA. After five days of co-culture, cells were harvested to detect ICOS^+^cTfh2 cells and plasma cells. Total IgG and anti-PvDBPII-specific antibodies were quantitated in culture supernatant.

### cTfh2 cell proliferation assay

To detect the proliferation of cTfh2 cells after their interaction with MBCs, the carboxyfluorescein succinimidyl ester (CFSE) proliferation assay was performed as previously described [27,30]. Briefly, PBMCs (5 x 10^6^) were labeled with 0.5 mM CFSE (Biolegend, USA) and the CFSE-positive naïve CD4^+^ T cells were sorted. The sorted cells were cultured for five days in the presence of anti-CD28/49d (10 ng/mL), IL-21 (20 ng/mL), IL-6 (20 ng/mL), and IL-4 (40 ng/mL) for generation of cTfh2-like cells. Then, autologous MBCs were added and stimulated with rPvDBPII (40 ng/mL) for 5 days. Positive controls of T-cell proliferation were cultures stimulated with 1% (v/v) PHA; negative controls were cultures without PvDBPII stimulation. The FlowJo TM LLC software version 10.10.0 (BD Biosciences, CA, USA) was used for the proliferation index analyses.

### Differentiation of plasma cells and IgG production

The levels of total IgG and anti-PvDBPII antibodies in co-culture supernatants were determined by ELISA as previously reported [31]. Briefly, 1 µg/mL anti-human IgG MT91/145 (Mabtech, Sweden) or 2 µg/mL of recombinant PvDBPII was coated on 96-well plates and blocked with phosphate-buffered saline with 10% fetal bovine serum (10% FBS-PBS). Undiluted culture supernatants were added to wells, followed by goat anti-human IgG conjugated to horseradish peroxidase (KPL, Milford, USA). Plates were developed with tetramethylbenzidine enzyme (TMB) substrate, and optical densities were read at 450 nm. Culture supernatants from PvDBPII-specific MBC cultures (A1F12) were used as positive controls [26]. Culture supernatants from non-PvDBPII stimulated B cells were used as background controls.

### Statistical analysis

Statistical analyses were conducted using GraphPad Prism software version 8.4.3 (GraphPad Software, USA, https://www.graphpad.com/). The non-parametric Mann-Whitney Rank Test was used to compare antibody responses and cTfh cells between *P.* vivax subjects and HCs or between HRs and LRs. The Wilcoxon matched-pairs signed rank test was used to assess the significant difference between PvDBPII stimulation and un-stimulation. One-way ANOVA followed by Dunn’s multiple comparison tests were used for multiple group comparisons in the proliferation and activation of cTfh2-like cells, as well as ASC differentiation and IgG secretion. Group differences with *p*-values < 0.05 were considered statistically significant. Correlation analysis was performed using Spearman’s rank correlation coefficient (*R*).

## Results

### Breadth and potency of antibodies against PvDBPII antigens in natural exposure

**Fig 1:**
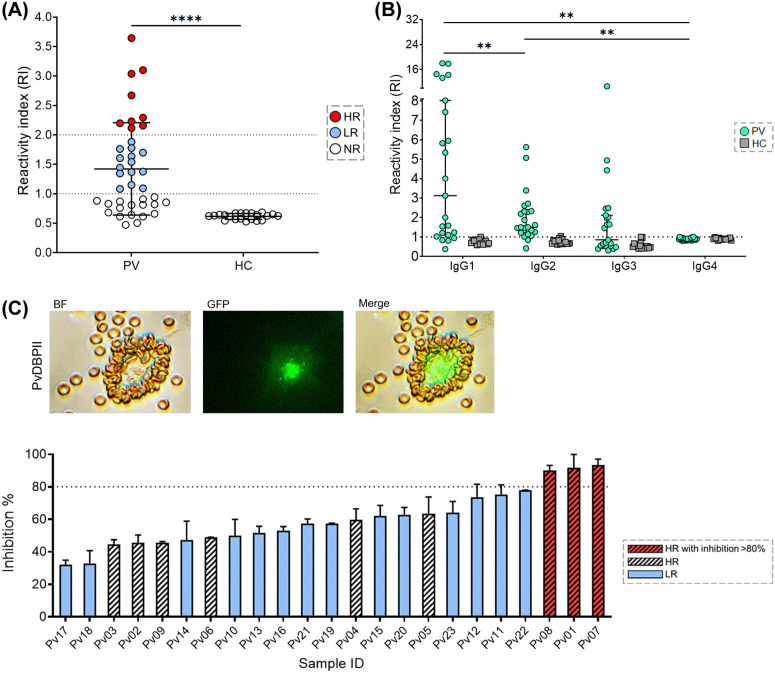
Seropositive responses and inhibitory function of anti-PvDBPII antibodies. **(A)** Total IgG levels against PvDBPII in plasma from acutely infected subjects (PV; n = 40) were determined and compared with healthy controls (HCs; n = 21). The total IgG reactivity index against PvDBPII is shown. The total IgG levels of seropositive responders were classified into high responders (HRs; ≥ cut-off + 2SD of the OD of HCs), low responders (LRs; > cut-off + 2SD of the OD of HCs). The seronegative samples were defined as non-responders (NRs; < cut-off of the OD of HCs). **(B)** Seropositive IgG *P. vivax* samples (n = 23) and HCs (n = 17) were used to assess IgG subclass (IgG1-4) responses against PvDBPII. The dashed line represents cut-off value of seropositivity (RI = 1) calculated from the mean of RI + 2SD of HC samples. Statistical testing was performed by Mann-Whitney Rank Test (for comparing two non-parametric groups; *p-value < 0.05, **p-value < 0.005, ****p-value < 0.0001. **(C) Top:** Representative images of PvDBPII binding to Duffy-positive human erythrocytes. Bright field (BF), GFP fluorescence, and merged pictures are shown. **Bottom:** Functional inhibition of plasma from acutely infected subjects seropositive for total IgG (n = 23) against PvDBPII–human erythrocyte binding. High-responder (HR) samples exhibiting > 80% inhibition are indicated by red stripes, HR samples with < 80% inhibition by black stripes, and low-responder (LR) samples by blue. Data are presented as mean ± SD of percentage inhibition.

To assess the inter-relationship of cTfh2 cells and humoral immunity during *P. vivax* infection, antibody titers and their inhibitory function were determined in acute *P. vivax* subjects. The anti-PvDBPII antibody titers in acute malaria subjects were significantly greater than those in HCs (p-value < 0.0001). Of the 40 subjects, 58% were seropositive for anti-PvDBPII IgG. Based on their anti-PvDBPII antibody levels, 22.5% (9/40), 35% (14/40), and 42.5% (17/40) of subjects were HRs, LRs, and NRs, respectively (Fig 1A). Further analysis of IgG subclass responses in the 23 seropositive subjects showed higher reactivity of cytophilic IgG1 (median 3.12 [IQR 1.10-8.021]) than to IgG2 (median 1.50 [IQR 1.21-2.33]), IgG3 (median 0.85 [IQR 0.53-2.1]), and IgG4 (median 0.88 [IQR 0.83-0.91]) (Fig 1B).

Next, the function of anti-PvDBPII antibodies to inhibit PvDBPII-erythrocyte binding was assessed using plasma samples from seropositive subjects (n = 23). Of the nine HR subjects, three (Pv01, Pv07 and Pv08) showed high inhibitory activity (91.68%, 93.40%, and 89.98%) (Fig 1C). None of the samples from LRs showed inhibition (< 80%) (Fig 1C).

### Upregulation of ICOS molecules on cTfh2 cells was observed in high responders in response to PvDBPII antigen

**Fig 2:**
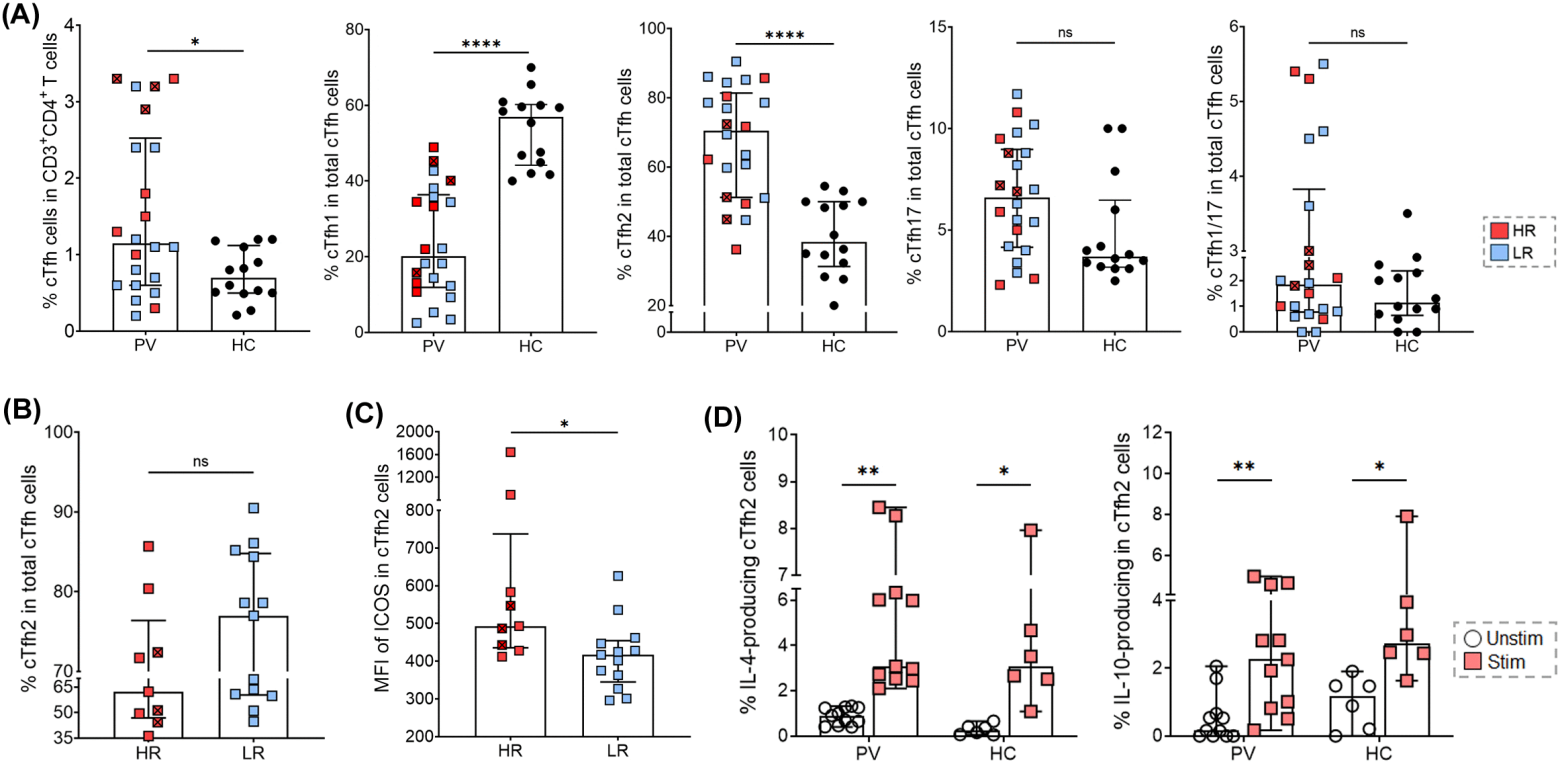
Frequency of cTfh2 cells in seropositive anti-PvDBPII antibody responses. PBMCs from acutely *P. vivax* infected (PV; n = 22) and healthy controls (HC; n = 14) were phenotyped by flow cytometry. **(A)** The frequencies of cTfh cells (CXCR5^+^ PD-1^+^) and cTfh subsets based on the expression of CXCR3 and CCR6 (cTfh1; CXCR3^+^CCR6^-^, cTfh2; CXCR3^-^CCR6^-^, cTfh17; CXCR3^-^CCR6^+^, and cTfh1/17; CXCR3^+^CCR6^+^). **(B)** The frequency of cTfh2 cells, comparing HR and LR subjects. (**C)** The MFI expression of ICOS molecules on the surface of cTfh2 cells was shown. **(D)** The frequency of IL-4- or IL-10-producing cTfh2 cells in *P. vivax* (n = 11) and healthy controls (HCs; n = 6). Each bar represents median, and error bar represents interquartile range (IQR). High-responder (HR) samples are shown in red, with > 80% inhibition indicated by red stripes, and low-responder (LR) samples in blue. Statistical testing was performed by Mann-Whitney Rank Test for comparing two non-parametric groups and Wilcoxon matched-pairs signed rank test; *p-value < 0.05; **p-value < 0.005; ***p-value < 0.0005; ****p-value < 0.0001; ns, non-significant.

To determine if the levels or inhibitory function of anti-PvDBPII antibodies correlate with cTfh2 cell function during *P. vivax* infection, we used flow cytometry to analyze the frequency of cTfh2 cells and their upregulation of costimulatory molecules (S1 Fig). The results were then compared between high and low antibody responders. During acute malaria, a significant expansion of cTfh cells was detected compared to the number in HCs (median 1.15 [IQR 0.57-2.4] vs median 0.55 [IQR 0.44-0.92], p-value < 0.05). Among four cTfh subsets, cTfh2 cells were markedly increased (median 70.55 [IQR 51.15-81.40] vs median 42.90 [IQR 31.23-50.00], p-value < 0.0001), whereas cTfh1 cells were significantly reduced (median 20.10 [IQR 11.90-36.35] vs median 51.50 [IQR 43.73-60.25], p-value < 0.0001). The frequencies of cTfh17 and cTfh1/17 cells during *P. vivax* infections did not differ significantly from those of HCs (cTfh17, median 6.60 [IQR 4.15-8.97] vs median 3.52 [IQR 2.98-7.3], p-value > 0.05; cTfh1/17, median 1.85 [IQR 0.77-3.82] vs median 1.14 [IQR 0.65-2.41], p-value > 0.05) (Fig 2A). Thus, expansions of cTfh2 cells in HRs and LRs were compared. The results showed that the frequencies of these cells in these two groups did not differ significantly (median 62.20 [IQR 46.85-76.40] vs median 77.00 [IQR 60.30-84.80], p-value > 0.05) (Fig 2B). Of the 23 seropositive samples, three subjects (Pv01, Pv07, and Pv08) produced high anti-PvDBPII antibody titers, and these individuals also showed high inhibitory titers and elevated cTfh2 cell frequencies (S2 Fig).

In addition, the functional phenotypes of cTfh2 cells were determined by assessing expression of ICOS, CD40L, and TIGIT co-stimulatory molecules on cells in HRs and LRs. Of the nine HRs, ICOS was significantly upregulated in comparison to that of LR subjects (median 482.0 [IQR 429.5-1267] vs median 417.0 [IQR 363.0-447.0], p-value = 0.012) (Fig 2C). However, there was no difference in expression of CD40L (median 184 [IQR 170.5-264.6] vs median 217.0 [IQR 108.0-340.0], p-value > 0.05) nor TIGIT between HRs and LRs (median 1002 [IQR 895.5-1048] vs median 893 [IQR 812.0-1010], p-value > 0.05) (S3 Fig). To further analyze the functional capacity of cTfh2 cells to produce IL-4 and IL-10, PBMCs from *P. vivax*-infected subjects who were anti-PvDBPII seropositive and from HCs were stimulated with PMA and ionomycin. Intracellular cytokine staining was performed to determine IL-4 and IL-10 expression, and results were compared with unstimulated cells (S4 Fig). The frequencies of IL-4- or IL-10-producing cTfh2 cells were significantly increased after stimulation in both *P. vivax* subjects (IL-4, median 3.08 [IQR 2.73-6.33] vs median 0.91 [IQR 0.46-1.26], p-value < 0.005; IL-10, median 2.46 [IQR 0.82-4.58] vs median 0.16 [IQR 0.00-0.66], p-value < 0.005) and HCs (IL-4, median 3.09 [IQR 2.15-5.48] vs median 0.26 [IQR 0.06-0.47], p-value < 0.05; IL-10, median 2.72 [IQR 2.23-4.94] vs median 1.18 [IQR 0.15-1.91], p-value < 0.05) (Fig 2D).

### Activation of cTfh2 cells triggered by *P. vivax* antigen

**Fig 3:**
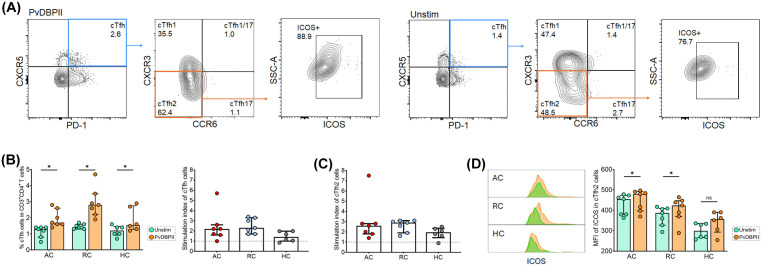
Activation of cTfh2 cells upon PvDBPII stimulation. PBMCs from acutely *P. vivax* infected subjects (AC; n = 7), recovered subjects (RC; n = 7), and healthy controls (HC; n = 6) were stimulated *in vitro* with recombinant PvDBPII (rPvDBPII) for 6 days. **(A).** Representative gating strategy for cTfh subsets between stimulated with rPvDBPII and un-stimulated (unstim). **(B)** The frequency of cTfh cells (CXC5^+^ PD-1^+^) and the Stimulation Index (SI) of cTfh cells after PvDBPII stimulation. **(C)** The SI of cTfh2 cells in culture of AC, RC, and HC subjects. **(D)** The representative of histogram and MFI ICOS expression on the surface of cTfh2 cells after *in vitro* culture with PvDBPII antigen. Each bar represents median, and error bar represents interquartile range (IQR). Statistical testing was performed by Wilcoxon matched-pairs signed rank test; *p-value < 0.05; ns, non-significant.

To further investigate whether activation of cTfh2 cells during infection was induced by *P. vivax* antigen, the *in vitro* stimulation of cell cultures was assessed. PBMCs from *P. vivax* subjects during acute and recovery phases of infection were stimulated with PvDBPII antigen. The frequency of cTfh cells and their cTfh2 subset as well as the activation of cTfh2 cells were observed (Fig 3A). After PvDBPII stimulation, significantly higher frequencies of cTfh cells were observed in cultures from AC, RC and HC subjects compared to those of unstimulated controls (AC: median 1.70 [IQR 1.60-2.60] vs. 1.30 [IQR 0.80-1.40], p-value < 0.05; RC: median 2.80 [IQR 2.20-3.50] vs. 1.40 [IQR 1.30-1.60], p-value < 0.05; HC: median 1.55 [IQR 1.47-2.75] vs. 1.20 [IQR 0.92-1.45], p-value < 0.05) (Fig 3B). The stimulation index (SI) of total cTfh cells in all subjects of AC and RC was greater than 1, indicating that cTfh cells were responsive to PvDBPII. We therefore sought to determine whether cTfh2 cells were activated following *P. vivax* antigen stimulation. The cTfh2 cells from all AC and RC subjects exhibited SI greater than 1 (Fig 3C). Moreover, these cTfh2 cells significantly upregulated ICOS expressions on their surfaces after PvDBPII stimulation (AC: median 480.0 [IQR 397.0-491.0] vs. 455.0 [IQR 380.0-473.0], p-value < 0.05; RC: median 424.0 [IQR 370.0-447.0] vs. 387.0 [IQR 327.0-409.0], p-value < 0.05) (Fig 3D).

### cTfh2-MBC interaction induced cTfh2 cell proliferation and activation

**Fig 4:**
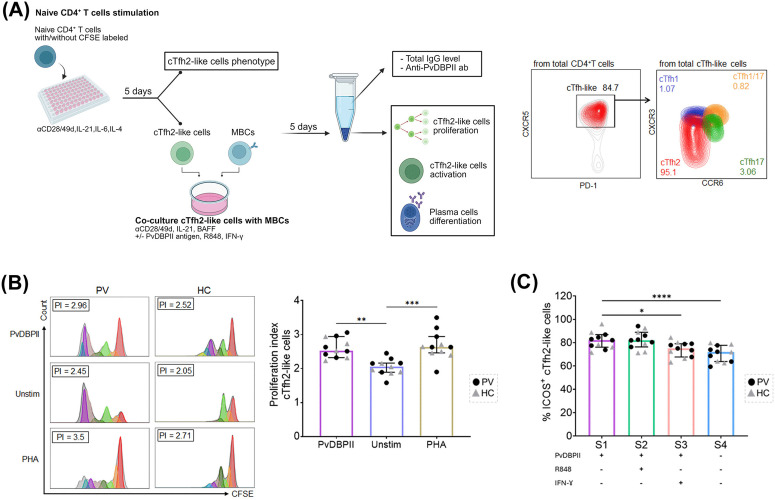
Proliferation and activation of the cTfh2-like cells after co-culture stimulation. **(A).** Experimental design: naïve CD4^+^T cells were sorted from *P. vivax* subjects (n = 6) and HCs (n = 5), labeled with or without CFSE, and then stimulated for 5 days to drive differentiation of cTfh2-like cells. The cTfh2-like cells were then co-cultured with autologous MBCs and the following stimuli: rPvDBPII antigen, R848 or IFN-γ. After 5 days, the cells were analyzed by flow cytometry to detect the proliferation and activation of cTfh2-like cells, along with the presence of ASCs. The culture supernatant was collected to detect total IgG and anti-PvDBPII antibody levels. The representative gating strategy for cTfh2 was based on CXCR3^-^CCR6^-^cTfh-like cells. **(B)** Representative proliferation of cTfh2-like cells under different conditions: PvDBPII stimulation, non-stimulation, or PHA stimulation. Data analysis was performed using FlowJo software, and the proliferation index was measured. **(C)** The frequency of ICOS^+^cTfh2-like cells in culture conditions: stimulation 1 (S1) rPvDBPII; S2) rPvDBPII and R848 stimulation; S3) rPvDBPII and IFN-γ; S4) No antigen stimulation (negative control). Positive control was PHA stimulation. Each bar represents the median, and the error bars represent the interquartile range (IQR). Statistical testing was performed by one-way ANOVA followed by Dunn’s multiple comparison test. Significance levels are as follows: *p-value < 0.05; **p-value < 0.005; ***p-value < 0.0005; ****p-value < 0.0001. The image was created in BioRender. Salsabila, Z. (2026) https://BioRender.com/np0qrx7.

To demonstrate the function of cTfh2 cells in helping MBCs to produce protective antibodies, samples from six HR seropositive subjects and five HCs were carried out in cTfh2-MBC co-cultures. We first established *in vitro* cultures to generate cTfh2-like cells (Fig 4A). Our results demonstrated that 82% to 95% of naïve CD4^+^T cells had differentiated into a cTfh2-like phenotype (CXCR3^-^CCR6^-^) during the culture period (Fig 4A). These cTfh2-like cells were utilized in a co-culture system with MBCs. Following PvDBPII stimulation, the proliferation of the differentiated cTfh2 cells showed a marked increase (median 2.52 [IQR 2.32-2.94] vs median 2.05 [IQR 1.89-2.16], p-value < 0.005), compared to those non-stimulated control (Fig 4B). We next investigated the activation of cTfh2 cells within a co-culture system. PvDBPII stimulation resulted in over-expression of ICOS molecules on the surface of these cTfh2 cells (PvDBPII stimuli, median 82.10 [IQR 76.10-86.90] vs un-stimulated, median 71.90 [IQR 63.80-77.70], p-value < 0.0001) (Fig 4C). The cTfh2-like cells showed no significant difference in ICOS upregulation following stimulation with PvDBPII and R848 (median 82.00 [IQR 76.30-88.90]) when compared to stimulation with PvDBPII alone (Fig 4C). In contrast, the presence of IFN-γ in co-cultures markedly decreased cTfh2 activation (median 75.10 [IQR 67.70-79.10], p-value < 0.05) (Fig 4C**).**

### cTfh2 cells drove MBC differentiation into ASCs and produced IgG antibodies

**Fig 5:**
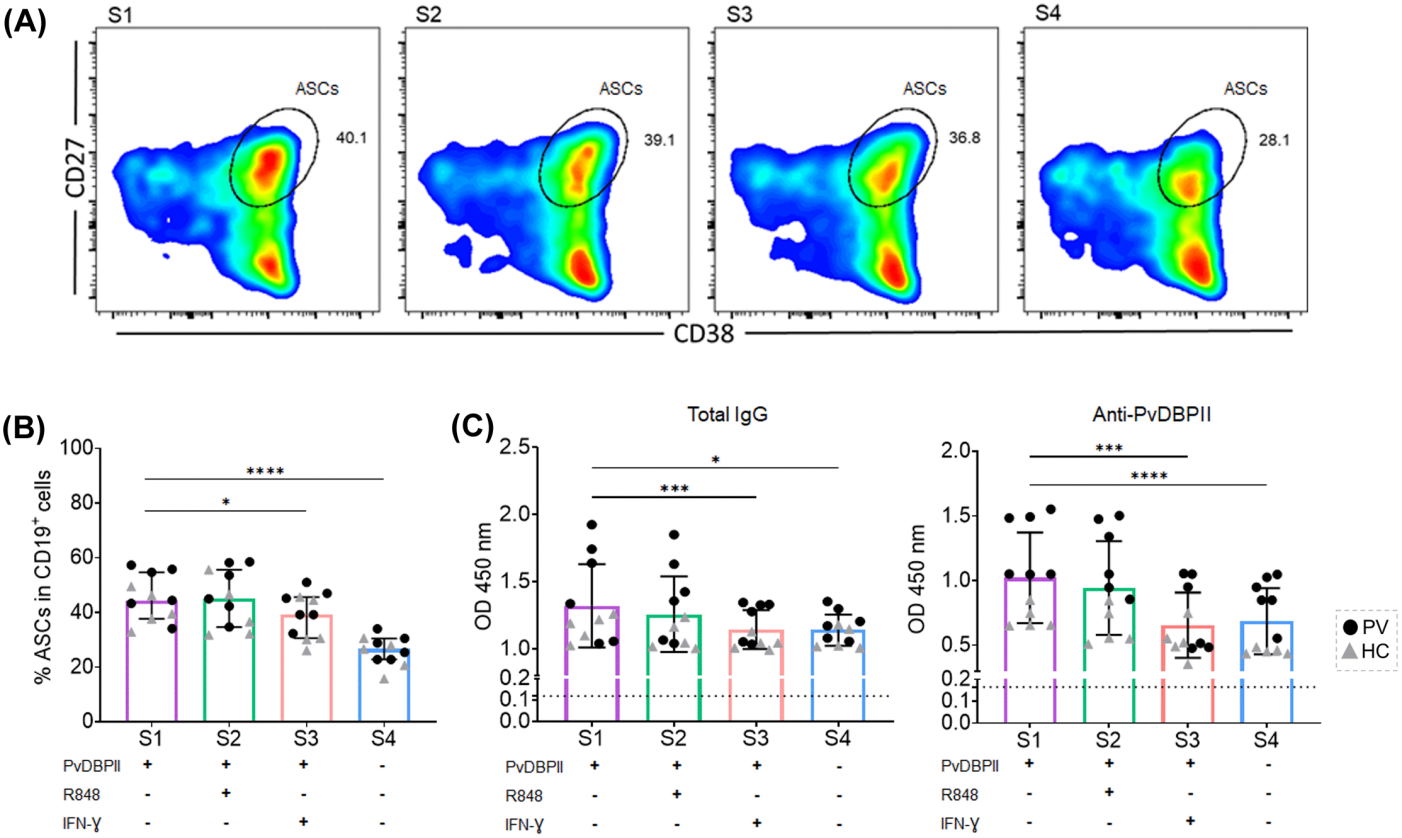
ASC differentiation and anti-PvDBPII antibody secretion after cTfh2-MBC co-cultures. **(A)** Representative gating of ASC differentiation based on CD19^+^CD27^+^CD38^+^ cells in cTfh2-like cell-MBC co-cultures with various stimulation conditions: S1) rPvDBPII stimulation, S2) R848 and rPvDBPII stimulation, S3) rPvDBPII and IFN-γ, S4) no antigen stimulation (negative control). **(B)** The frequency of ASCs after co-culture. **(C)** Total IgG and anti-PvDBPII antibody levels were measured in culture supernatants. Each bar represents median, and error bar represents interquartile range (IQR). Statistical testing was performed by one-way ANOVA analysis and multiple-comparison by Dunn’s multiple comparison; *p-value < 0.05; **p-value < 0.005; ***p-value < 0.0005; ****p-value < 0.0001.

To assess the capability of cTfh2 cells to drive MBCs to differentiate into ASCs and secrete antibodies, cTfh2-like cells produced *in vitro* were co-cultured with autologous MBCs. The frequency of ASCs (CD27^+^CD38^+^) was then measured after rPvDBPII stimulation (Fig 5A). Concurrently, co-culture supernatants were harvested to quantify the levels of total IgG and anti-PvDBPII antibodies. A significant increase of ASCs was observed compared to non-stimulated co-cultures (median 44.40 [IQR 37.70-54.70] vs median 26.60 [IQR 22.80-30.50], p-value < 0.0001) (Fig 5B). Furthermore, marked increases in both total IgG (median 1.21 [IQR 1.06-1.64] vs median 1.14 [IQR 1.02-1.20], p-value < 0.05) and anti-PvDBPII antibodies were detected in the co-culture supernatant following PvDBPII stimulation relative to non-stimulated conditions (median 1.05 [IQR0.65-1.48] vs median 0.55 [IQR 0.44-0.94], p-value < 0.0001) (Fig 5C).

We next assessed the capacity of TLR7/8 and IFN-γ signals to stimulate cTfh2-MBC function. Addition of the agonist R848 to co-cultures did not further increase ASC differentiation (median 45.00 [IQR 34.70-55.60]) nor antibody secretion (anti-PvDBPII, median 0.85 [IQR 0.55-1.34]), when compared to PvDBPII stimulation alone (Fig 5B and 5C). Addition of IFN-γ to co-cultures led to significant reductions of ASC differentiation and IgG secretion, compared to PvDBPII stimulation (p-value < 0.005) (Fig 5B and 5C).

## Discussion

The relative importance of different Tfh cell subsets in antibody induction appears to be specific to the disease, pathogen and/or vaccine context. In malaria, cTfh2 cells are implicated in MBC responses and antibody production [17,18]. However, it is currently unclear how cTfh cells help the development of humoral immunity during malaria infection. Here, we demonstrated the phenotypic characteristics and function of cTfh2 cells to promote MBC responses following natural *P. vivax* infections. Expansions of cTfh2 cells were detected in PvDBPII-seropositive subjects. In a comparison between High and Low Responders, the activated ICOS^+^cTfh2 cells were higher in HRs, while there was no difference in the overall cTfh2 frequency. Notably, a few HRs had high frequencies of cTfh2 cells and produced inhibitory antibodies against PvDBPII binding to erythrocytes. The cTfh2 cells were specifically stimulated by *P. vivax* antigen, which led to their proliferation and activation. Subsequently, they promoted B cell differentiation into antibody-secreting cells and antibody secretion. Together, these data provide insights into how cTfh2 cells orchestrate humoral immune responses during *P. vivax* infection, highlighting their contribution to helping B cells produce anti-malarial antibodies.

The elevated frequency of cTfh2 cells in malaria is associated with specific antibody responses to the infection [16,18,19]. The production of anti-PfMSP2 IgG and IgM antibodies during acute malaria is significantly correlated with the frequency of cTfh2 cells [16]. Additionally, a recent study of vivax malaria subjects identified positive correlations among cTfh2 cells, anti-PvDBPII antibodies and MBCs [18]. These previous findings indicate the contribution of cTfh2 cells to the development of anti-malarial humoral immunity. However, a knowledge gap remains regarding the mechanisms by which malaria-specific cTfh2 cells are generated, and how their interaction with B cells promotes development of protective antibodies against malaria. In this study, we enrolled subjects with acute *P. vivax* infection to assess the titer and inhibitory function of their antibodies, and the frequency of cTfh2 cells. Of the 40 infected subjects, 14 were defined as High and nine as Low Responders. The frequency of cTfh2 cells did not differ between these groups, whereas activated ICOS^+^cTfh2 cells were more frequent in HRs, including three (Pv01, Pv07 and Pv08) who showed cTfh2 cell activation and produced inhibitory antibodies that blocked PvDBPII binding to erythrocytes. We sought to assess the effect of age and prior malaria exposure on cTfh2 cells and functional antibodies in these subjects. Subject Pv01 (age > 60 years) had a re-infection with *P. vivax*, while subjects Pv07 and Pv08 (ages < 39 years) had no history of prior malaria. Since only a few samples produced functional antibodies, our data could not establish an association between cTfh2 cells and the production of protective antibodies in *P. vivax* malaria. A deep investigation into the molecular levels of cTfh2 cells from malaria subjects who produced functional antibodies against parasite invasion, compared to non-responders, could be helpful in understanding mechanisms of cTfh2 cells in helping the development of anti-malarial protective immunity.

To demonstrate whether the activation of cTfh2 cells during acute malaria is specifically triggered by a parasite antigen, PBMCs from the acute and recovery phases of *P. vivax* infection were stimulated with PvDBPII antigen. A significant increase in the frequency of CXCR5^+^PD-1^+^cTfh cells was observed upon *in vitro* stimulation with rPvDBPII. Further phenotyping of these expanded cells revealed the cTfh2 subset phenotype. These findings indicated that *P. vivax* exposure initially triggered cTfh2 cell responses, showing an expansion of these cells during acute malaria, and that subsequently, upon re-stimulation with the parasite antigen, these cells were activated and carried out their function. Our data aligns with a previous study in Brazil that showed the ability of *P. vivax*-infected reticulocytes to induce cTfh cell responses [17]. Similarly, a study of *P. falciparum* malaria demonstrated an increase in cTfh2 activation following *in vitro* stimulation with malaria-infected red blood cells [16]. However, the responses of cTfh cell subsets to a specific malaria antigen are significantly influenced by age and prior parasite exposures in endemic areas. For example, adults with falciparum malaria produced cTfh17 and cTfh1/17 cell responses to glutamic acid-rich protein (GARP), while children produced a broader response of cTfh subsets (cTfh1, cTfh2, cTfh17 and cTfh1/17) after GARP and schizont egress antigen-1 (SEA-1A) stimulation [14]. Repeated antigen exposure may boost malaria-specific memory cTfh cells, as PvDBPII re-stimulation *in vitro* significantly increased the activation of cTfh2 cells. These findings likely reflect the presence of expanded effector memory cTfh2 cells during acute malaria [18] which can rapidly reactivate and polarize toward functionally specialized subsets. Collectively, our findings support the concept that exposure to *P. vivax* drives a change toward distinct cTfh cell responses, particularly the cTfh2 subset, in specific antibody production. A deeper understanding of mechanisms to drive polarization of malaria-specific cTfh2 cells for their function should be useful in malaria vaccine design for antibody targeting.

The interaction between Tfh and B cells promotes MBC development for the generation of long-lived plasma cells [32–35]. Here, we demonstrated a co-operative function between cTfh2 and B cells in vivax malaria using a co-culture system. The cTfh2-like cells were produced from naïve CD4^+^T cells upon cytokine stimulation with IL-4, IL-6 and IL-21. In the cTfh2-B cell co-cultures, we first determined that signals from MBCs and PvDBPII antigen induced the proliferation and activation of cTfh2 cells. The coordinated interactions between cTfh2 and MBCs (via the ICOS-ICOSL signal) drove the process of MBC differentiation into ASCs and the secretion of antibodies. Signals received from both BCR and TLR-7/8 ligands stimulated cTfh2 cell activation, but no more than stimulation with PvDBPII alone. These results are similar to those of a study in *P. yoelii* NSM-infected mice showing that a TLR7 agonist and the lysate of infected red blood cells induced splenic Tfh cell activation and differentiation [36]. Based on our findings, the signal from TLR-7/8 ligand-stimulated MBCs is not the sole stimulus for enhancing cTfh2 cell function. A combination of other signals that activate specific transcription factors like GATA3 and c-Maf, as well as the IL-21 cytokine, may also be required to promote the differentiation and function of cTfh2 cells [12,37–39]. Concerning the effect of the IFN-γ on cTfh2 function, our data showed a reduction in cTfh2-like cell activation after adding IFN-γ to co-culture stimulation, which subsequently led to decreases of ASC differentiation and antibody secretion. These findings are consistent with previous observations that Plasmodium-induced cTfh1 cytokine responses (IFN-γ, IL-12p70 and TNF-α) are associated with the activation of cTfh1-like CXCR3^+^memory cells that exhibit impaired B cell function [15]. Together, our results suggest a potential role of cTfh2-MBC interaction for anti-malaria antibody secretion. Future strategies for malaria vaccine design should target Tfh2 cells to stimulate MBC function and the production of long-lasting antibodies against malaria parasites.

It should be noted that there were limitations in this study. Only a few samples of *P. vivax* showed an expansion of cTfh2 cells and produced functional antibodies against PvDBPII-human erythrocyte binding. A large sample size will be needed to confirm the factors involved in cTfh2 cell activation and its contribution to the development of functional anti-malarial antibodies. Since antigen specificity is a critical factor in triggering the response of a particular subset of cTfh cells [14,16], our data demonstrated that the blood-stage antigen PvDBPII induced cTfh2 cell function. More investigation into different *P. vivax* antigens could help to generate deep knowledge of cTfh2 cell function in *P. vivax* malaria. Additionally, the limited number of cTfh2 cells in circulating blood makes it challenging to demonstrate co-operative functions between cTfh2 and B cells using *in vitro* systems. Here, naïve CD4^+^T cells from *P. vivax* malaria subjects were differentiated into cTfh2 cells (cTfh2-like cells) before being added to co-cultures with MBCs. Our results demonstrated that an interaction between cTfh2 and MBCs generated anti-*P. vivax* specific antibodies.

## Supporting information

S1 Table Demographic information of *P*. *vivax* subjects and malaria naïve healthy donors recruited in this study. (DOCX)

S2 Table List of fluorochrome conjugated antibodies. (DOCX)

S1 Fig Gating strategy of cTfh2 phenotype. cTfh cells were distinguished by CXCR5 and PD-1 T cells (CD3^+^CD4^+^CD45RA^-^). cTfh cell subsets were distinguished by CXCR3 and CCR6 as cTfh1 (CXCR3^+^CCR6^-^), cTfh2 (CXCR3^-^CCR6^-^), cTfh17 (CXCR3^-^CCR6^+^), and cTfh1/17 (CXCR3^+^CCR6^+^). The functional phenotypes of cTfh2 cells were based on ICOS^,^ CD40L, and TIGIT molecules.
(TIF)

S2 Fig Correlation analysis of antibody titers, inhibitory function and frequency of cTfh2 cells in PvDBPII seropositive subjects. The correlation was performed by Spearman correlation. The straight line represents the trend of correlation. Spearman ρ and p-value for each correlation are indicated.
(TIF)

S3 Fig The expression of CD40L and TIGIT molecules on the surface of cTfh2 cells. The MFI expression of CD40L and TIGIT was compared between HR and LR subjects.
(TIF)

S4 Fig Gating strategy of IL-4 or IL-10 producing cTfh2 cells. cTfh cells were distinguished by CXCR5 and PD-1 T cells (CD3^+^CD4^+^). cTfh cell subsets were distinguished by CXCR3 and CCR6 as cTfh1 (CXCR3^+^CCR6^-^), cTfh2 (CXCR3^-^CCR6^-^), cTfh17 (CXCR3^-^CCR6^+^), and cTfh1/17 (CXCR3^+^CCR6^+^). The IL-4 and IL-10 were then gated from cTfh2 cell subset.
(TIF)

S1 File Raw data for Figs 1–5. (XLSX)
