# Supplementary material for: A functional crosstalk between circulating follicular helper 2 T cells and memory B cells drives anti-Plasmodium vivax antibodies
Source: PLoS Negl Trop Dis. 2026 Apr 24;20(4):e0014232. doi: 10.1371/journal.pntd.0014232 (PMC13108860; doi:10.1371/journal.pntd.0014232)
Supplement: S1 Table — (DOCX) [file pntd.0014232.s001.docx]

**S1 Table. Demographic information of *P*. *vivax* subjects and malaria naive healthy donors recruited in this study.**

| **Characteristics** | **Acute *P. vivax* patients** | **Recovered**  ***P. vivax*  patients**  **(7–8 months)** | **Healthy controls** |
| --- | --- | --- | --- |
| **Total Number (n)** | 40 | 7 | 21 |
| **Age (years)** |  |  |  |
| Median (IQR) | 41 (26.25-60.75) | 45 (32.00-61.00) | 29 (25.50-36.50) |
| **Gender** |  |  |  |
| Male | 62.5% (25/40) | 57.14% (4/7) | 57% (12/21) |
| Female | 37.5% (15/40) | 42.85% (3/7) | 43% (9/21) |
| **Nationality** |  |  |  |
| Thai | 90% (36/40) | 100% (7/7) | 100% (21/21) |
| Myanmar | 10% (4/40) | 0% | 0% (0/21) |
| **No. of prior infection** |  |  |  |
| 0 | 39 | 7 | 21 |
| 1 | 1 | 0 | 0 |
| **Parasitemia (parasite/µl)** |  |  |  |
| Mean ± SD (range) | 1512.60 ± 1610.59  (16.00-5577) | 0 | 0 |
