# Supplementary material for: A functional crosstalk between circulating follicular helper 2 T cells and memory B cells drives anti-Plasmodium vivax antibodies
Source: PLoS Negl Trop Dis. 2026 Apr 24;20(4):e0014232. doi: 10.1371/journal.pntd.0014232 (PMC13108860; doi:10.1371/journal.pntd.0014232)
Supplement: S2 Table — (DOCX) [file pntd.0014232.s002.docx]

**S2 Table. List of fluorochrome conjugated antibodies.**

| **Reagent or resource** | **Source** | **Identifier** | **Additional information** |
| --- | --- | --- | --- |
| Antibodies | | | |
| CD3-AlexaFlour700 | BioLegend | Cat#317340; RRID:AB_2563408 | 1:100 dilution |
| CD4-PerCP/Cy5.5 | BioLegend | Cat#317428; RRID:AB_1186122 | 1:200 dilution |
| CXCR5-PE | BioLegend | Cat#356904; RRID:AB_2561813 | 1:50 dilution |
| PD-1(CD279)-APC | BioLegend | Cat#329908; RRID:AB_940475 | 1:50 dilution |
| CXCR3-APC/FIRE750/Cy7 | BioLegend | Cat#353722; RRID:AB_2561423 | 1:50 dilution |
| CCR6-PE/Cy7 | BioLegend | Cat#353418; RRID:AB_10916518 | 1:20 dilution |
| ICOS-AlexaFlour 488 | BioLegend | Cat#313514; RRID:AB_2122584 | 1:50 dilution |
| CD40L(CD154)-BV421 | BioLegend | Cat#310824; RRID:AB_2562721 | 1:50 dilution |
| TIGIT-BV605 | BioLegend | Cat#372712; RRID:AB_2632927 | 1:50 dilution |
| CD3-PerCP/Cy5.5 | BioLegend | Cat#300430; RRID:AB_893299 | 1:100 dilution |
| CD4-FITC | BioLegend | Cat#317408; RRID:AB_571951 | 1:100 dilution |
| CD45RA-PE | BioLegend | Cat#304108; RRID:AB_314412 | 1:50 dilution |
| CD19-FITC | BioLegend | Cat#302206; RRID:AB_314236 | 1:100 dilution |
| CD27-PE/Cy7 | BioLegend | Cat#356412; RRID:AB_2562258 | 1:50 dilution |
| IgD-PerCP/Cy5.5 | BioLegend | Cat#348208; RRID:AB_10641706 | 1:50 dilution |
| CD27-APC/FIRE750/Cy7 | BioLegend | Cat#356428; RRID:AB_2616711 | 1:50 dilution |
| CD38-AlexaFlour700 | BioLegend | Cat#356624; RRID:AB_2566424 | 1:200 dilution |
| IL-4-BV711 | BioLegend | Cat#500848; RRID:AB 3097266 | 1:20 dilution |
| IL-10-PE/Dazzle 594 | BioLegend | Cat#501426; RRID:AB_2566744 | 1:25 dilution |
